# Sex disparities in the effect of statins on lipid parameters: The PharmLines Initiative

**DOI:** 10.1097/MD.0000000000028394

**Published:** 2022-01-14

**Authors:** Nicholas B. Hunt, Johanna E. Emmens, Sylvi Irawati, Stijn de Vos, Jens H.J. Bos, Bob Wilffert, Eelko Hak, Rudolf A. de Boer

**Affiliations:** a Groningen Research Institute of Pharmacy, PharmacoTherapy, -Epidemiology & -Economics, University of Groningen, Groningen, The Netherlands; b Division of Pharmacoepidemiology & Clinical Pharmacology, Utrecht Institute for Pharmaceutical Sciences (UIPS), Utrecht University, Utrecht, The Netherlands; c Department of Cardiology, University of Groningen, University Medical Center Groningen, Groningen, The Netherlands; d Centre for Medicines Information and Pharmaceutical Care, Faculty of Pharmacy, Universitas Surabaya, Surabaya, Indonesia; e Department of Clinical and Community Pharmacy, Faculty of Pharmacy, Universitas Surabaya, Surabaya, Indonesia; f University of Groningen, Department of Clinical Pharmacy & Pharmacology, University Medical Center Groningen, Groningen, The Netherlands.

**Keywords:** drug prescriptions, lipids, medical record linkage, pharmacoepidemiology, sex, statins, treatment outcome

## Abstract

Real-world evidence on a potential statin effect modification by sex is inconclusive, especially for the primary prevention of cardiovascular disease (CVD). We aimed to quantify the differences in the effect of statins on lipid parameters between men and women.

The PharmLines Initiative linked the Lifelines Cohort Study and the IADB.nl prescription database. This database covers a representative population from the Netherlands. We selected participants aged ≥40 years at the index date: the date of the first prescription of any statin monotherapy in the study period 2006 to 2017. Multivariate regression modeling was used to compare the difference of the mean percentage change of lipid parameters (% mean difference [MD]) from baseline to follow-up measurement between the sexes.

Out of 5366 statin users from approximately 50,000 participants available in the final linked database, 685 were statin initiators. At baseline, women had significantly higher levels of mean total cholesterol (TC), low-density lipoprotein cholesterol (LDL-C), and high-density lipoprotein cholesterol (HDL-C) than men (all *P* values <.01). At follow-up, women had a significantly higher mean percentage change of HDL-C compared to men (adjusted % MD 5.59, 95% confidence interval [CI] 2.42-8.75, *P* < .01). There was no significant sex difference in other parameters, nor in the proportion of men and women who achieved LDL-C ≤2.5 mmol/L.

Statins appear to have a greater effect on increasing HDL-C levels in women than men while showing similar effect on other lipid parameters in both sexes. Men should not be treated differently than women.

## 1 Introduction

In Europe, cardiovascular disease (CVD) contributes to 40% and 49% of all deaths in men and women, respectively. It burdens 79% of European countries with 40 to 150 disability-adjusted life years per 1000 citizens.^[1,2]^ Statins are the primary lipid-lowering agents recommended by guidelines from the American Heart Association/American College of Cardiology, the European Society of Cardiology, and the Dutch College of General Practitioners to prevent CVD.^[3–6]^ The clinical benefit of statins is mainly due to its ability to reduce low-density lipoprotein cholesterol (LDL-C) concentration. In general, statins should be prescribed for individuals with a 10-year moderate to high risk of developing CVD (primary prevention) based on a total cardiovascular (CV) risk assessment as well as LDL-C concentration, and individuals with established CVD (secondary prevention). These guidelines present different scoring systems to assess an individual's total CV risk and provide a separate scoring chart for men and women.^[3,5–7]^ Despite this, there are no sex-specific guidelines for statin therapy.

Two meta-analyses of randomised controlled trials of statins vs control (placebo/less-intensive dose) showed no sex disparities in the effect of statins on reducing major CV events.^[8,9]^ One review showed the difference in the effects of statins for primary and secondary CVD prevention between sexes to be inconsistent.^[10]^ When it comes to sex disparities in the effect of statins on lipid parameters, meta-analyses show disagreement. One shows that the mean absolute reduction of LDL-C after 1-year of using statin is significantly greater in men than in women. However, for total cholesterol (TC), high-density lipoprotein cholesterol (HDL-C) and triglycerides (TG), this effect was similar between sexes.^[8]^ In the other meta-analysis, women experience a more significant reduction in LDL-C, but a less significant increase in HDL-C, than men.^[11]^

Studies using real-world data mostly detect sex disparities in CV risk assessment, statin administration, adherence, and adverse effects.^[10,12–15]^ They offer limited explanation of the sex disparities in lipid modification, especially for primary prevention.^[10,16]^ We aimed to investigate disparities in the effectiveness of statins on important lipid parameters between women and men who were first time users of statins for both the primary and secondary prevention of CVD in a real-world setting.

## 2 Methods

We report our study according to the REporting of studies Conducted using Observational Routinely-collected health Data statement for pharmacoepidemiology.^[17]^

### 2.1 Study design and setting

We conducted an inception cohort study using the PharmLines Initiative database that linking data from the Lifelines Cohort Study and the IADB.nl prescription database. The overall design of the Lifelines Cohort Study, the IADB.nl prescription database, and the Pharmlines Initiative have been described elsewhere.^[18–22]^

Lifelines is a population-based database established to investigate the contribution of socio-demographic, physical, psychological, biomedical, and behavioural factors to the development of disease and health of general population living in the North of the Netherlands.^[18,20,22]^ IADB.nl is a population-based database that has been prospectively collecting prescription data from community pharmacies in the Netherlands since 1996. As in 2017, the coverage of the IADB.nl is around 700,000 participants from approximately 70 community pharmacies.^[19,21]^

IADB.nl supplies full prescription data regardless of health insurance status. It has been extensively used for research and has been found to represent the whole Netherlands in terms of age, sex, and prescription rates. The information stored in the IADB.nl relevant to this study such as the date of birth and sex of each participant, the date of medication being dispensed, the quantity of medication, the dose of medication (in terms of defined daily dose, DDD), and the number of days of valid prescription. Each medication is registered according to the Anatomical Therapeutic Chemical code. The database however records neither medications bought over the counter by the participants nor medications dispensed in the hospital. To maintain confidentiality, a unique anonymous identifier is given to every participant and used to track each participant's prescription record throughout the database.^[19,21]^

The Lifelines study protocol is approved by the medical ethical committee of the University Medical Center Groningen and all Lifelines participants have each signed an informed consent stating that they approve the use of their (anonymized) data and material for scientific purposes. Data of the IADB.nl is collected according to the national and European guidelines on privacy with human data valid at the time of collection.

### 2.2 Database linkage

Briefly, the linking process was the responsibility of the trusted third-party, the Netherlands’ Central Agency for Statistics (Centraal Bureau voor de Statistiek). The linkage was performed at the individual level based on combined information of 4-digit postal code, sex, and date of birth. A new unique identifier, which could not be tracked back to identifier in the individual databases, then was assigned to each participant.^[21]^

### 2.3 Study participants, compared groups, outcomes, and follow-up

**Figure 1:**
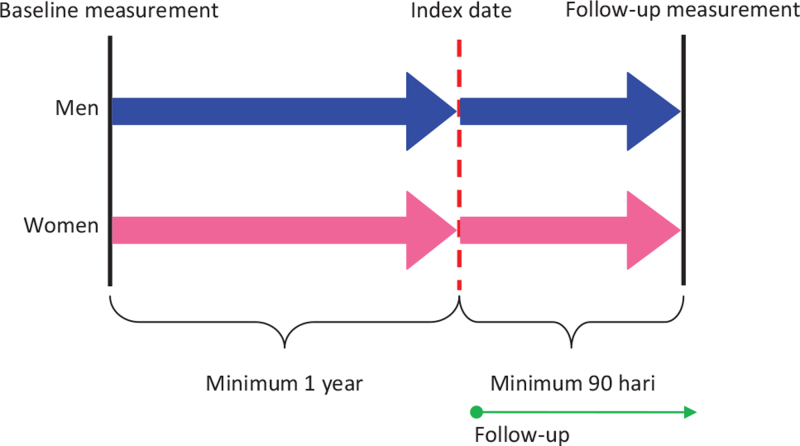
Design of the retrospective inception cohort study.

We included participants ≥40 years of age at the index date, defined as the date of the first prescription of any statin monotherapy (Anatomical Therapeutic Chemical code C10AA) during the study period (2006-2017). Statin monotherapy was determined by an absence of other lipid-lowering agents at index date. Participants were only included if they were present in the database for at least 365 days before the first prescription of any statins and had both the baseline and follow-up visit recorded in the Lifelines database. Participants were excluded if they had used statins for less than 90 days (Fig. 1).

We further classified the statin initiators into 2 groups: initiators for primary prevention and initiators for secondary prevention of CVD. For the primary prevention group, participants were excluded if they had previously been diagnosed with CVD, as defined by the algorithm developed by van der Ende et al,^[18]^ including the diagnoses of myocardial infarction, cerebrovascular accident, transient ischemic attack, aortic aneurysm, or peripheral artery disease. Men were the reference group for all outcome comparisons.

Our primary outcome was the sex difference in the mean percentage change (% mean difference, %MD) of TC, LDL-C, HDL-C, and TG level from baseline to follow-up and in the achievement of LDL-C treatment target (≤2.5 mmol/L), as recommended by the 2011 Dutch guidelines, for the all-statin initiator group,^[4,23]^ As described previously, Roche Modular P automated analyzer (Mannheim, Germany) was used to measure lipid parameters. The plasma cholesterol used in clinical chemistry analyses was obtained from blood veins after an overnight fast. TC, LDL-C, and HDL-C were measured with direct enzymatic colorimetric assays whereas TG was measured with an assay based on glycerol phosphate oxidase-peroxidase aminophenazone. All assays were standardized. Friedewald formula was used to calculate LDL-C.^[24]^

As secondary outcomes, we measured the sex differences in the effect of statins separately for primary and secondary prevention and in participants’ adherence to statins. Adherence was calculated as the proportion days covered where the number of days covered with statin prescriptions were divided by the number of days between index date and follow-up multiplied by 100. Participants were classified as adherent when proportion days covered was ≥80%.^[25]^

### 2.4 Statistical analyses

Proportions for categorical variables, mean ± standard deviation for normally distributed continuous variables, and median and interquartile range for skewed continuous variables are reported. Chi-square tests, independent sample *t* tests, and Mann–Whitney *U* tests were used to compare categorical variables, normally-distributed continuous variables, and skewed variables, respectively. The distribution of variables were determined using P-P, Q-Q plots and stem and leaf plots, where outliers were identified and subsequently removed. A complete case analysis was performed to account for any sporadically missing data in the confounder and outcome variables. A potential for collinearity between dependent and independent variables were examined before the linear regression analyses were performed. We looked at the Pearson correlation score (*r*) and the variance inflation factor (VIF) to detect multicollinearity. The presence of multicollinearity was suggested when *r* > 0.90 and VIF score >10.^[26]^

We report %MD ± standard errors from linear regression, odds ratios from logistic regression, and their 95% confidence intervals (95% CI). Statistically significant co-variables (*P* < .05) in univariate analysis were included in multivariate linear and logistic regression analyses. IBM Statistical Package for Social Sciences Statistics 22 (IBM Corp., Armonk, N.Y., USA) was used to perform all statistical analyses.

## 3 Indirect patient and public involvement

Patients and public were involved in the development of the Lifelines database. Patient representatives were involved in the updating of the database.

## 4 Results

**Figure 2:**
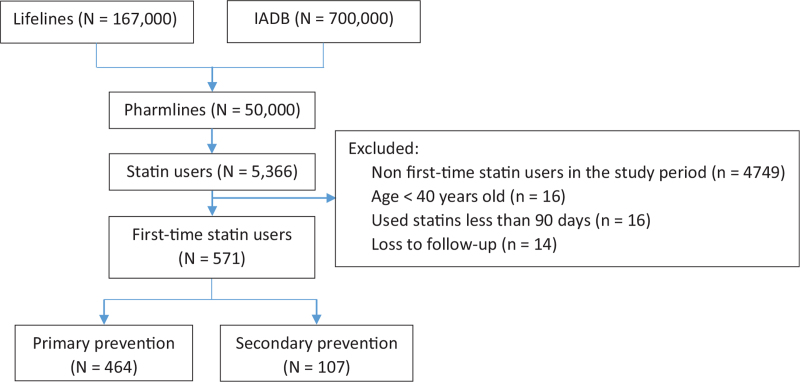
Flow diagram of the selection of participants.

Out of around 50,000 participants available in the linked database, 5366 were statin users. Of these, 571 participants were first time statin users in the study period. Among these participants, 282 (49.4%) were men and 464 (81.3%) had initiated statins for primary prevention (Fig. 2). The year of the Lifelines baseline appointments ranged from 2006 to 2013 and the Lifelines follow-up appointments ranged from 2014 to 2017. Between these 2 periods, the time of statin initiations ranged from May 11, 2006 to August 4, 2016. The overall mean duration between the baseline measurement date and the index date was 710.66 ± 638 days. The overall mean duration of follow-up was 928.97 ± 484.70 days. Simvastatin was used the most by both men (91.5%) and women (90.0%).

Compared to men, women were significantly older, and had higher levels of most lipid parameters including TC, LDL-C, and HDL-C at baseline (Table 1). Men had significantly higher mean systolic and diastolic blood pressure. There were no differences in mean body mass index, smoking status, the presence of diabetes and hypercholesterolemia, and the mean starting dose of statins between the sexes at baseline. However, although the mean duration of follow-up between the sexes was not significantly different, the median of follow-up in women was significantly longer than in men.

**Table 1:** Baseline characteristics of the all statin initiator group.

	Men (N = 282)	Women (N = 289)	
Variables (unit)	Mean ± SD	Mean ± SD	*P* value
Age (yrs)	53 (48, 64)^∗^	57 (49, 66)^∗^	.072
BMI (kg/m^2^)	27.44 ± 3.47	27.74 ± 4.85	.400
SBP (mm Hg)	137.70 ± 16.01	131.87 ± 18.18	**<.001**
DBP (mm Hg)	81.25 ± 9.94	75.14 ± 9.09	**<.001**
Baseline lipid parameters			
TC (mmol/L)	5.96 ± 1.12	6.36 ± 1.17	**<.001**
LDL-C (mmol/L)	4.08 ± 1.02	4.33 ± 1.09	**.004**
HDL-C (mmol/L)	1.26 ± 0.31	1.50 ± 0.42	**<.001**
TG (mmol/L)	1.93 ± 1.72	1.69 ± 1.11	.055
Starting dose of statins (mg)^†^			
Simvastatin	34.14 ± 9.59 (n = 258)	32.50 ± 10.81 (n = 260)	.067
Atorvastatin	23.33 ± 11.13 (n = 15)	30 ± 12.40 (n = 14)	.139
Duration of follow-up (d)	844.50 (508.5, 1209)^∗^	978.00 (585, 1263)^∗^	**.017**
Cardiovascular risk factors	n (%)	n (%)	
Current smokers	42 (14.89)	42 (14.53)	.918
Hypertension	94 (33.33)	118 (40.83)	**.032**
Hypercholesterolemia	81 (28.72)	91 (31.49)	.255
Diabetes mellitus	11 (3.90)	15 (5.19)	.312

BMI = body mass index, DBP = diastolic blood pressure, HDL-C = high-density lipoprotein cholesterol, LDL-C = low-density lipoprotein cholesterol, N = number of participants included in the analysis, n = number of participants with the displayed variable, SBP = systolic blood pressure, SD = standard deviation, TC = total cholesterol, TG = triglycerides.

∗ Median (25^th^, 75^th^ percentiles).

† Pravastatin and rosuvastatin were not included in the analysis because they were used by less than 10 participants in 1 or both groups.

### 4.1 Sex disparities in the effect of statins on lipid parameters

After adjustments for potential confounders, in both men and women separately, statins significantly decreased the levels of TC and LDL-C, and increased the level of HDL-C from baseline to follow-up (Table 2). However, there was a more significantly improved HDL-C level in women compared to men in the adjusted pairwise comparison (adjusted MD 5.64%, 95% CI 2.36-8.92, *P* < .01), the differences in the mean percentage change of TC, LDL-C, and TG from baseline between the sexes were not statistically significant. The proportion of men and women who attained the LDL-C treatment target was similar, only 37% for both groups. The adherent rates were moderate (73.1% in men and 72.0% in women) and also similar between the sexes (Table 3).

**Table 2:** Comparison of the effect of statins between the sexes on lipid parameters in the all statin initiator group.

		Unadjusted				Adjusted			
Lipid parameters (mmol/L)	Groups	N	MD ± SE (%)	95% CI	*P* value	N	MD ± SE (%)	95% CI	*P* value
TC	Sex difference	543	1.13 ± 1.56	−1.93, 4.18	.470	542	−0.43 ± 1.56	−2.63, 3.48	.784^∗^
	Men	272	−20.99 ± 1.02	−23.15, −18.83		272	−21.80 ± 1.05	−23.85, −19.74	
	Women	271	−22.11 ± 1.17	−24.40, 19.82		270	−21.37 ± 1.05	−23.43, −19.31	
LDL-C	Sex difference	543	2.36 ± 2.34	−2.24, 6.97	.315	542	−0.61 ± 2.36	−5.25, 4.03	.797^†^
	Men	272	−26.05 ± 1.44	−28.87, −23.23		272	−27.58 ± 1.59	−30.70, −24.46	
	Women	271	−28.41 ± 1.85	−32.04. −24.78		270	−26.97 ± 1.59	−30.10, −23.84	
HDL-C	Sex difference	543	−2.14 ± 1.56	−5.21, 0.93	.171	542	−5.64 ± 1.67	−8.92, −2.36	**.001** ^†^
	Men	272	5.74 ± 1.03	3.32, 7.35		272	3.59 ± 1.12	1.38, 5.79	
	Women	271	7.47 ± 1.18	5.17, 9.77		270	9.23 ± 1.13	7.02, 11.44	
TG	Sex difference	543	−8.32 ± 4.00	−16.18, −0.45	**.038**	542	7.60 ± 4.24	0.73, 15.92	.073^†^
	Men	272	−2.56 ± 3.38	−9.19, 4.07		272	−2.89 ± 2.85	−8.48, 2.70	
	Women	271	−10.87 ± 2.14	−15.06, 6.68		270	−10.49 ± 2.86	−16.10, 4.88	

CI = confidence interval, DBP = diastolic blood pressure, HDL-C = high-density lipoprotein cholesterol, LDL-C = low-density lipoprotein cholesterol, MD = mean difference, N = number of participants included in the analysis, n = number of participants with the displayed lipid parameter, SBP = systolic blood pressure, SE = standard error, TC = total cholesterol, TG = triglycerides.

∗ Adjusted for age, SBP, DBP, TC, HDL-C, TG, and starting dose of simvastatin at baseline.

† Adjusted for age, SBP, DBP, LDL-C, HDL-C, TG, and starting dose of simvastatin at baseline.

**Table 3:** Comparison of the effect of statins on the achievement of treatment goal and adherence to statins between the sexes in the all statin initiator group.

Outcomes	Men (n/N, %)	Women (n/N, %)	OR (95% CI; *P* value)
Achieving treatment goal (LDL-C ≤ 2.5 mmol/L)	105/282, 37.2%	101/289, 37.4%	Crude: 1.01 (0.72, 1.41; .970)
			Adjusted: 1.22 (0.82, 1.82; .322)^∗^
Adherence to statins (PDC ≥ 80%)	206/282, 73.1%	208/289, 72.0%	Crude: 0.96 (0.66, 1.39; .830)
			Adjusted: 0.96 (0.64,1.46; .854)^∗^

CI = confidence interval, DBP = diastolic blood pressure, HDL-C = high-density lipoprotein cholesterol, LDL-C = low-density lipoprotein cholesterol, N = number of participants included in the analysis, n = number of participants with the outcome variable, OR = odds ratio, PDC = proportion days covered, SBP = systolic blood pressure, TG = triglycerides.

∗ Adjusted for age, SBP, DBP, LDL-C, HDL-C, TG, and starting dose of simvastatin at baseline.

In line with the all-statin initiator group results, statin use in the primary and secondary prevention subgroups, were found to increase the HDL-C level to a significantly greater extent in women than in men (all *P* values < .05; primary prevention: adjusted MD 4.82%, 95% CI 1.10-8.54; secondary prevention: adjusted MD 8.79%, 95% CI 1.66, 15.93; Table S1, Supplemental Digital Content). There were no significant differences between the sexes in the mean percentage change from baseline for other lipid parameters, the achievement LDL-C treatment goal, or adherence to statin therapy both in subgroups of primary and secondary prevention (Table S2, Supplemental Digital Content and Table S3, Supplemental Digital Content).

## 5 Discussion

In all statin users, we found a significantly greater mean percentage increase in HDL-C concentration after initiating statin therapy in women compared to men and no statistically significant differences between the sexes regarding the other lipid parameters. Remarkably, the proportion of men and women who achieved the LDL-C treatment goal was below 40% without statistically significant differences between the sexes. In the primary prevention group the level of attainment of LDL-C treatment target was even lower than 35% for both sexes. However, in the secondary prevention group, the proportion of men and women who reached the treatment target was above 45%, although the differences were not significant between the sexes. Despite the low rate of achievement of the treatment target, the level of adherence to statins was 70% in both sexes.

Our findings contradict results from the meta-analysis of clinical trials by Karlson et al^[11]^ where statins led to a significantly greater increase of 0.5% in the HDL-C mean percentage from baseline in men compared to women. Additionally, this meta-analysis found a significantly greater decrease of 2.1% in the LDL-C mean percentage from baseline in women compared to men.^[11]^ On the other hand, in agreement with our findings, the Cholesterol Treatment Trialist’ Collaboration's meta analysis demonstrated similar trend of statin effects on the change in mean percentages of TC, LDL-C, and HDL-C from baseline to 1-year follow up between the sexes.^[8]^

The more significant effect of statins to raise HDL-C in women than in men despite the small sample size in our study is an interesting finding. HDL-C response to statins has been investigated in an individual participant meta-analysis of clinical trials in the VOYAGER database.^[11,27]^ There was a significant low-to-moderate correlation between the change in HDL-C percentage and the change in the TG percentage, both from baseline to follow-up, induced by statin therapy. The greater the reduction in TG percentage, the greater the increase in HDL-C percentage. However, this study did not differentiate whether there was a difference of this phenomenon between men and women.^[27]^ In our study there was a trend toward greater decrease in TG level in women compared to men, though statistically nonsignificant, was accompanied by a greater increase of HDL-C level in women compared to men. The underlying mechanism of this relationship is unclear.

Low baseline HDL-C and high baseline TG were found as independent predictors of a higher percentage change of HDL-C from baseline for atorvastatin, rosuvastatin, and simvastatin.^[27,28]^ Women in our study had a higher baseline HDL-C and a lower baseline TG compared to men, yet they still demonstrated a greater HDL-C response to statins. The extent of HDL-C elevation also depends on the type and dose of statins.^[27–30]^ Rosuvastatin (5-40 mg) led to 5.5% to 7.9% increase of HDL-C concentration in a direct dose-dependent relationship whereas atorvastatin (10-80 mg) changed HDL-C level in an inverse dose-dependent relationship (4.5% at the 10 mg to 2.3% at the 80 mg). Simvastatin (10-80 mg) raised HDL-C by 4.2% to 5.3% in a similar fashion to rosuvastatin.^[27]^

HDL-C response may also depend on the type of patients. In Chinese diabetic patients, atorvastatin, younger age (<65 years), body mass index ≥24 kg/m^2^ and women with baseline HDL-C >1.29 mmol/L or men with baseline HDL-C >1.03 mmol/L predicted a decrease of HDL-C level after 1-year of statin therapy. Severe atherogenic dyslipidemia (baseline TG ≥2.30 mmol/L and HDL-C ≤0.88 mmol/L), but not women with TG >1.69 mmol/L and HDL-C ≤1.29 mmol/L or men with HDL-C ≤1.03 mmol/L, were protective factors against HDL-C decrease in these patients.^[29]^ In our study, other factors might oppose the HDL-C elevating effect of statins in men.

The level of adherence to statin therapy in men (73.1%) and women (72.0%) in our study is considered moderate and similar whereas the proportion of participants who achieved the LDL-C treatment goal is below 40%. These results are consistent with other studies. A recent systematic review (2019) of 16 published studies investigating predictors of statin adherence found that the level of adherence to statin therapy for primary and/or secondary prevention was suboptimal (range: 41.0%-82.7%).^[31]^ One study using the PHARMO, a general practitioner database in the Netherlands, showed that from all population treated with statins on average 1 daily defined dose, 45% did not reached the LDL-c treatment target according to the guidelines. Our study found a lower LDL-C treatment goal attainment although the actual filled-prescription of the drug by the patients could be assessed in the PharmLines database whereas it was not available in the PHARMO database.^[32]^

Our study provides evidence on the possible differences in the effectiveness of statins between men and women in a real-world setting. Which is especially important for primary prevention, where the current evidence is lacking. The whole population of the Netherlands and the adult population of the North of the Netherlands are each well represented by the data from IADB.nl and Lifelines, respectively.^[19,33]^ The recruitment strategy means the selection bias is low that the results obtained from Lifelines can be applied to the general population.^[33]^

Our study might lack statistical power to detect smaller differences between sexes due to a relatively small sample size included in the analysis. Only 1% of participants in the final linked database initiated statins between their 2 Lifelines appointments and performing a complete-case analysis contributed to a low precision, notably in subgroups. A lack of information on in-hospital dispensed medications in the IADB database might cause a small number of participants in the secondary prevention group. As Lifelines follow-up is still ongoing and the IADB is ever-evolving and expanding, repeating this study in the future should yield results with higher statistical power.

There still remains uncertainty surrounding the potential sex differences in the effectiveness of statins. The literature presents a varied picture, but here we find the effects of statins on TC, LDL-C, and TG between the sexes are similar whereas HDL-C response appears to be higher in women than men. This difference could be due to other factors than statin type or dose or adherence which oppose the HDL-C elevating effect of statins in men. The degree to which an increase of HDL-C level corresponds to a reduction in CV major events needs further investigation. In all, the results are compatible with the fact that men should not be treated different with statins than women.

## Acknowledgments

The authors wish to acknowledge the services of the Lifelines Cohort Study, the contributing research centres delivering data to Lifelines, and all the study participants, and the participating IADB.nl pharmacies for kindly providing their data for research.

## Author contributions

NBH, JEE, EH, and RAdB contributed to the conception or design of the work. SdV contributed to the statistical analysis. All authors contributed to the acquisition, analysis, or interpretation of the data. NBH, JEE and SI drafted the manuscript. All authors critically revised the manuscript, gave final approval, and agreed to be accountable for all aspects of work ensuring integrity and accuracy.

**Conceptualization:** Nicholas B. Hunt, Johanna E. Emmens, Eelko Hak, Rudolf A. de Boer.

**Data curation:** Nicholas B. Hunt, Sylvi Irawati.

**Formal analysis:** Nicholas B. Hunt, Johanna E. Emmens, Sylvi Irawati, Stijn de Vos, Eelko Hak, Rudolf A. de Boer.

**Methodology:** Nicholas B. Hunt, Johanna E. Emmens, Eelko Hak, Rudolf A. de Boer.

**Project administration:** Nicholas B. Hunt, Johanna E. Emmens, Eelko Hak.

**Resources:** Jens H.J. Bos, Bob Wilffert, Eelko Hak, Rudolf A. de Boer.

**Software:** Jens H.J. Bos.

**Supervision:** Bob Wilffert, Eelko Hak, Rudolf A. de Boer.

**Validation:** Nicholas B. Hunt, Johanna E. Emmens, Sylvi Irawati, Jens H.J. Bos, Eelko Hak, Rudolf A. de Boer.

**Writing – original draft:** Nicholas B. Hunt.

**Writing – review & editing:** Nicholas B. Hunt, Johanna E. Emmens, Sylvi Irawati, Stijn de Vos, Jens H.J. Bos, Bob Wilffert, Eelko Hak, Rudolf A. de Boer.

## Supplementary Material

Supplemental Digital Content

## Supplementary Material

Supplemental Digital Content

## Supplementary Material

Supplemental Digital Content
