# Supplementary material for: Sex disparities in the effect of statins on lipid parameters: The PharmLines Initiative
Source: Medicine (Baltimore). 2022 Jan 14;101(2):e28394. doi: 10.1097/MD.0000000000028394 (PMC8758030; doi:10.1097/MD.0000000000028394)
Supplement: Supplemental Digital Content [file medi-101-e28394-s001.docx]

**Supplementary material Table 1**

**Table 1.** Comparison of the effect of statins on lipid parameters between the sexes in the primary and secondary prevention group.

| Lipid parameters (mmol/L) | Subgroups |  | Unadjusted | | | | Adjusted | | | |
| --- | --- | --- | --- | --- | --- | --- | --- | --- | --- | --- |
|  |  |  | N^a^ | MD ± SE (%) | 95% CI | p-Value | N^a^ | MD ± SE (%) | 95% CI | p-Value |
| TC | Primary prevention | Sex difference | 441 | 1.39 ± 1.69 | -4.70, 1.92 | 0.410 | 440 | -0.313 ± 1.67 | -2.97, 3.59 | 0.851^b^ |
|  |  | Men | 218 | -20.91 ± 1.08 | -23.03, -18.79 |  | 218 | -21.81 ± 1.13 | -24.03, -19.60 |  |
|  |  | Women | 223 | -22.30 ± 1.29 | -24.82, -19.78 |  | 222 | -21.50 ± 1.12 | -23.69, -19.31 |  |
|  | Secondary prevention | Sex difference | 102 | -0.044 ± 3.97 | -7.83, 7.92 | 0.991 | 102 | -0.978 ± 4.21 | -7.38, 9.34 | 0.817^b^ |
|  |  | Men | 54 | -21.30 ± 2.76 | -26.71, -15.89 |  | 54 | -21.74 ± 2.74 | -27.17, -16.31 |  |
|  |  | Women | 48 | -21.25 ± 2.84 | -26,81, -15.69 |  | 48 | -20.76 ± 2.92 | -25.56, -14.96 |  |
| LDL-C | Primary prevention | Sex difference | 441 | -2.74 ± 2.57 | -7.80, 2.31 | 0.287 | 440 | -1.09 ± 2.58 | -3.99, 6.16 | 0.674^c^ |
|  |  | Men | 218 | -26.07 ± 1.53 | -29.08, -23.06 |  | 218 | -28.06 ± 1.74 | -31.49, -24.63 |  |
|  |  | Women | 223 | -28.81 ± 2.05 | -32.83, -24.79 |  | 222 | -26.97 ± 1.73 | -30.37, -23.58 |  |
|  | Secondary prevention | Sex difference | 102 | -0.579 ± 5.74 | -11.96, 10.80 | 0.920 | 102 | 0.37 ± 6.01 | -12.30, 11.56 | 0.951^c^ |
|  |  | Men | 54 | -26.00 ± 3.84 | -33.53, -18.47 |  | 54 | -26.10 ± 3.91 | -33.86, -18.33 |  |
|  |  | Women | 48 | -26.58 ± 4.28 | -34.97, -18.19 |  | 48 | -26.47 ± 4.18 | -34.76, -18.18 |  |
| HDL-C | Primary prevention | Sex difference | 441 | - 1.06 ± 1.75 | - 2.38, 4.51 | 0.544 | 440 | -4.82 ± 1.89 | -8.54, -1.10 | **0.011**^c^ |
|  |  | Men | 218 | 6.63 ± 1.16 | 4.37, 8.89 |  | 218 | 4.73 ± 1.28 | 2.22, 7.24 |  |
|  |  | Women | 223 | 7.69 ± 1.13 | 5.12, 10.26 |  | 222 | 9.55 ± 1.27 | 7.07, 12.04 |  |
|  | Secondary prevention | Sex difference | 102 | -6.34 ± 3.36 | -0.32, 13.00 | 0.062 | 102 | -8.79 ± 3.59 | -15.93, -1.66 | **0.016**^c^ |
|  |  | Men | 54 | 0.11 ± 2.13 | -4.06, 4.28 |  | 54 | -1.04 ± 2.34 | -5.69, 3.61 |  |
|  |  | Women | 48 | 6.45 ± 2.64 | 1.28, 11.62 |  | 48 | 7.75 ± 2.50 | 2.79, 12.71 |  |
| TG | Primary prevention | Sex difference | 441 | 6.22 ± 4.17 | -14.42, 1.97 | 0.136 | 440 | 6.87 ± 4.40 | -1.78, 15.52 | 0.119^c^ |
|  |  | Men | 218 | -5.00 ± 3.40 | -11.67, 1.67 |  | 218 | -4.64 ± 2.97 | -10.48, -1.20 |  |
|  |  | Women | 223 | -11.22 ± 2.43 | -15.99, -6.45 |  | 222 | -11.51 ± 2.94 | -17.29, -5.73 |  |
|  | Secondary prevention | Sex difference | 102 | 16.55 ± 11.40 | -39.17, 6.07 | 0.150 | 102 | 6.93 ± 12.15 | -31.06, 17.20 | 0.570^c^ |
|  |  | Men | 54 | 7.28 ± 10.06 | -12.44, 27.00 |  | 54 | 2.75 ± 7.91 | -12.96, 18.46 |  |
|  |  | Women | 48 | -9.27 ± 4.24 | -17.58, 0.96 |  | 48 | -4.18 ± 8.44 | -20.95, 12.59 |  |

^a^Number of participants included in the analysis

^b^Adjusted for age, SBP, DBP, TC, HDL-C, TG, and starting dose of simvastatin at baseline

^c^Adjusted for age, SBP, DBP, LDL-C, HDL-C, TG, and starting dose of simvastatin at baseline

CI, confidence interval; DBP, diastolic blood pressure; HDL-C, high-density-lipoprotein cholesterol; LDL-C, low-density lipoprotein cholesterol; MD, mean difference; SBP, systolic blood pressure; SE, standard error; TC, total cholesterol; TG, triglycerides
