# Supplementary material for: Sex disparities in the effect of statins on lipid parameters: The PharmLines Initiative
Source: Medicine (Baltimore). 2022 Jan 14;101(2):e28394. doi: 10.1097/MD.0000000000028394 (PMC8758030; doi:10.1097/MD.0000000000028394)
Supplement: Supplemental Digital Content [file medi-101-e28394-s002.docx]

**Table 2.** Comparison of the effect of statins on the achievement of treatment goal (LDL-C ≤ 2.5 mmol/L) between the sexes in the primary and secondary prevention group.

| Treatment groups | Men (n/N, %) | Women (n/N, %) | Crude OR | 95% CI | p-Value | Adjusted OR^a^ | 95% CI | p-Value |
| --- | --- | --- | --- | --- | --- | --- | --- | --- |
| Primary prevention | 79/226, 34.9 | 80/238, 33.6 | 0.94 | 0.64, 1.38 | 0.761 | 1.11 | 0.71, 1.74 | 0.659 |
| Secondary prevention | 26/56, 46.4 | 28/51, 54.9 | 1.41 | 0.66, 3.01 | 0.382 | 2.02 | 0.81, 5.03 | 0.131 |

^a^Adjusted for age, SBP, DBP, LDL-C, HDL-C, TG, and starting dose of simvastatin at baseline

CI, confidence interval; DBP, diastolic blood pressure; HDL-C, high-density-lipoprotein cholesterol; LDL-C, low-density lipoprotein cholesterol; OR, odds ratio; SBP, systolic blood pressure; TG, triglycerides
