# Supplementary material for: Sex disparities in the effect of statins on lipid parameters: The PharmLines Initiative
Source: Medicine (Baltimore). 2022 Jan 14;101(2):e28394. doi: 10.1097/MD.0000000000028394 (PMC8758030; doi:10.1097/MD.0000000000028394)
Supplement: Supplemental Digital Content [file medi-101-e28394-s003.docx]

**Table 3.** Comparison of the adherence to statins between the sexes in the primary and secondary prevention group.

|  | Men (n/N, %) | Women (n/N, %) | Crude OR | 95% CI | p-Value | Adjusted OR^a^ | 95% CI | p-Value |
| --- | --- | --- | --- | --- | --- | --- | --- | --- |
| Primary prevention | 164/226, 72.5 | 173/238, 72.6 | 1.01 | 0.67, 1.51 | 0.976 | 1.02 | 0.64, 1.62 | 0.935 |
| Secondary prevention | 42/56, 75.0 | 35/51, 68.6 | 0.73 | 0.31, 1.70 | 0.464 | 0.70 | 0.27, 1.83 | 0.464 |

^a^Adjusted for baseline age, SBP, DBP, LDL-C, HDL-C, TG, and starting dose of simvastatin

CI, confidence interval; DBP, diastolic blood pressure; HDL-C, high-density-lipoprotein cholesterol; LDL-C, low-density lipoprotein cholesterol; OR, odds ratio; PDC, proportion days covered; SBP, systolic blood pressure; TG, triglycerides
